# Diagnostic Accuracy of Adenosine Deaminase and Lymphocyte Proportion in Pleural Fluid for Tuberculous Pleurisy in Different Prevalence Scenarios

**DOI:** 10.1371/journal.pone.0038729

**Published:** 2012-06-18

**Authors:** Alberto Garcia-Zamalloa, Jorge Taboada-Gomez

**Affiliations:** 1 Mycobacterial Infection Study Group (GEIM) from the Spanish Infectious Diseases Society, Department of Internal Medicine, Mendaro Hospital, Mendaro, Gipuzkoa, Spain; 2 Preventive Medicine and Western Gipuzkoa Clinical Research Unit, Mendaro Hospital, Mendaro, Gipuzkoa, Spain; Public Health Agency of Barcelona, Spain

## Abstract

**Background:**

Tuberculous pleural effusion (TPE) is a paucibacillary manifestation of tuberculosis, so isolation of *Mycobacterium tuberculosis* is difficult, biomarkers being an alternative for diagnosis. Adenosine deaminase (ADA) is the most cost-effective pleural fluid marker and is routinely used in high prevalence settings, whereas its value is questioned in areas with low prevalence. The lymphocyte proportion (LP) is known to increase the specificity of ADA for this diagnosis. We analyse the diagnostic usefulness of ADA alone and the combination of ADA ≥40 U/l (ADA_40_) and LP≥50% (LP_50_) in three different prevalence scenarios over 11 years in our area.

**Materials and Methods:**

Biochemistry, cytology and microbiology studies from 472 consecutive pleural fluid samples were retrospectively analyzed. ADA and differential cell count were determined in all samples. We established three different prevalence periods, based on percentage of pleural effusion cases diagnosed as tuberculosis: 1998–2000 (31.3%), 2001–2004 (11.8%), and 2005–2008 (7.4%). ROC curves, dispersion diagrams and pre/post-test probability graphs were produced. TPE accounted for 73 episodes (mean prevalence: 15.5%). The sensitivity, specificity, positive predictive value (PPV) and negative predictive value (NPV) for ADA_40_ were 89%, 92.7%, 69.2% and 97.9%, respectively. For ADA_40_+LP_50_ the specificity and PPV increased (98.3% and 90%) with hardly any decrease in the sensitivity or NPV (86.3% and 97.5%). No relevant differences were observed between the three study periods.

**Conclusions/Significance:**

ADA remains useful for the diagnosis of TPE even in low-to-intermediate prevalence scenarios when combined with the lymphocyte proportion.

## Introduction

Tuberculous pleural effusion (TPE) is a common manifestation of extrapulmonary tuberculosis, and is the leading cause of pleural effusion in developing world regions, while it is much less common in developed countries [1]–[3].

The diagnosis of TPE depends on the demonstration of tubercle bacilli in pleural fluid, a pleural biopsy specimen or sputum, or the demonstration of granulomas in the pleura [4]. Due to the paucity of *Mycobacterium tuberculosis* in the pleural fluid, the performance of a pleural biopsy has historically been considered the most reliable method to confirm the diagnosis when tuberculous aetiology of a pleural effusion is suspected. However, since pleural tissue sampling is more difficult than simple thoracocentesis, pleural fluid markers of TPE have been extensively evaluated as an attractive alternative to pleural biopsy [5]. ADA is the most cost-effective pleural fluid marker and is routinely employed as a screening tool, in particular, in countries where tuberculosis is endemic [1].

Theoretically, according to Bayes theorem, the predictive value of a marker such as ADA depends not only on its sensitivity and specificity, but also on the local prevalence of the disease: in a high prevalence setting the positive predictive value (PPV) of elevated ADA would increase, while in a low prevalence setting the PPV would decline but the negative predictive value (NPV) would remain high, so a low concentration of ADA might rule out TPE [1], [5]. On the other hand, the combination of ADA and the pleural fluid lymphocyte proportion (LP) has come to be recognised as an excellent approach for increasing the specificity of ADA test [6].

The aim of this research was to assess the accuracy of ADA combined with pleural fluid LP for TPE in our area, in three different prevalence settings over a total period of eleven years.

## Methods

We retrospectively reviewed all consecutive patients with pleural effusion who underwent a diagnostic thoracocentesis at Mendaro Hospital (community hospital, which provides healthcare to Bajo Deba Area - 80,000 inhabitants - in Basque Country, Spain) from January 1998 to December 2008. The ADA level and LP in pleural fluid had been determined in all samples. We recorded demographic and clinical data (body temperature on admission, and presence of cough, chest pain and/or dyspnoea), and pleural fluid data (total and differential cell count, protein, lactate dehydrogenase-LDH, pH, glucose, ADA, cytology, aerobic and anaerobic culture, Lowenstein-Jensen and MGIT-Bactec culture), along with serum glucose, protein and LDH. Histopathological and microbiological findings in pleural biopsy specimens, final diagnosis and, in TPE cases, first and last day of treatment and the combination of anti-tuberculosis drugs given were also recorded. When more than one thoracocentesis was performed, the statistical analysis was performed using only the data from the first pleural fluid sample, although the outcomes of every sample were recorded.

Regarding the diagnostic criteria, two subclasses of TPE were identified: 1) confirmed pleural tuberculosis (CPTE): identification of the bacillus in pleural fluid, sputum or pleural biopsy by stain or by culture; or by the presence of granulomatous inammation on pleural biopsy, and 2) probable pleural tuberculosis (PTPE): clinical and radiological evidence for tuberculosis in the absence of any other obvious cause associated with pleural effusion and positive response to a complete course of anti-tuberculosis treatment, confirmed through outpatient follow-up of at least twelve months. Malignant effusion was diagnosed when malignant cells were found in pleural fluid or pleural biopsy. Uncomplicated parapneumonic effusion (UPE) referred to any exudative effusion associated with bacterial pneumonia, lung abscess or bronchiectasis which resolved with antibiotics alone, while patients with pH <7.20 or glucose <40 mg/dl or Gram’s stain or culture positive were classed as having complicated parapneumonic effusion (CPE), with empyema describing the presence of frank pus in the pleural cavity [7]. Within the miscellaneous group of diagnoses we included other types of pleural effusion with different aetiologies and defined by well established clinical criteria. Transudative effusions were defined following Light’s criteria [8]. Finally, there was a last group for patients with pleural effusion in whom an exact diagnosis had not been reached.

For the purposes of this study, following the approach of previous authors, “prevalence” was used to refer to the number of cases of a given type of pleural effusion divided by the total number of pleural effusions studied, more appropriately named “pre-test probability” [1], [5], [9].

Pleural fluid ADA levels were determined by using an automated ultraviolet kinetic assay (Roche Diagnostics, Mannheim, Germany). White blood cells were manually counted in a Thoma chamber, and differential cell counts in pleural fluid were performed after Wright’s staining by counting, whenever possible, 200 cells. The LP was calculated as the number of lymphocytes divided by the total number of white cells.

Statistical analysis was performed using SPSS 18.0 statistical software. Categorical variables were analyzed with the χ^2^ test and continuous variables were compared using the Student’s t-test. In addition, whenever parametric tests could not be used, Kruskal–Wallis one-way analysis of variance was performed to compare group means. Assessing the performance with the ROC curve, we explored the usefulness of a single parameter relating the ADA levels to the proportion of lymphocytes; specifically, we multiplied the level of ADA by the LP, calling the resulting new variable ADA·LP. Diagnostic accuracy, including 95% confidence intervals, assessed using sensitivity, specificity, predictive values, likelihood ratios and area under the ROC curve (AUC) in the tuberculosis and non-tuberculosis sub-groups. ROC curves were constructed to compare ADA with ADA·LP. Whenever appropriate, Pearson’s correlation coefficient was also calculated.

This research counts with the permission of the CEO of the Mendaro Hospital and the approval of the Western Gipuzkoa Research Commision for the development of the study.

## Results

A total of 472 episodes of pleural effusion were analysed, with a male:female ratio of 294∶178 cases (62.3%:37.7%). Mean age was 66.2 years (SD of 20.25 years).

Overall, there were 73 cases of TPE (15.4%), 29 of CPE or empyema (6.1%), 92 of UPE (19.5%), 105 of malignant effusion (22.2%), 78 of miscellaneous exudates (16.5%), 61 of transudative effusion (12.9%) and 34 of undiagnosed pleural effusion (7.2%).

Among the 73 episodes of TPE, 25 cases were confirmed to be pleural tuberculosis (CTPE) (19 by positive culture and 6 by presence of granulomas in pleural biopsy tissue) and the remaining 48 cases were probable pleural tuberculosis (PTPE), given the clinical symptoms combined with biochemical and cytological results from pleural fluid and response to a complete course of treatment.

ADA activity was significantly higher in CTPE, PTPE and CPE/empyema (Table 1). Specifically, using the Kruskal-Wallis one-way analysis of variance, we found statistically significant differences in mean ADA levels between the CTPE, PTPE and CPE/empyema group and the other diagnostic entities: UPE, malignant effusions, miscellaneous exudates, transudative and undiagnosed effusions. Differences between tuberculosis (confirmed or probable) and CPE/empyema were not significant. Nevertheless, CTPE and PTPE were easily distinguished from CPE/empyema by the pleural fluid white cell count: mean LP in TPE and CPE/empyema being 83.3% and 16%, respectively. In Table 2 we report the number and percentage of positive results obtained for the two diagnostic criteria used: an ADA level equal to or greater than 40 U/l (ADA_40_) and a LP equal to or greater than 50% (LP_50_), by type of pleural effusion. Although in six out of the 25 patients with CTPE the ADA level was lower than 40 U/l in the first pleural fluid sample, a subsequent thoracocentesis yielded an ADA level higher than 40 U/l in four cases.

**Table 1:** ADA levels by type of pleural effusion in three tuberculosis pleural effusion prevalence periods (1998–2000, prevalence: 31.3%; 2001–2004, prevalence: 11.8%; and 2005–2008, prevalence: 7.4%).

	Whole period		1998–2000		2001–2004		2005–2008	
	Mean	Standard Deviation	Mean	Standard Deviation	Mean	Standard Deviation	Mean	Standard Deviation
CTPE	56.7	28.2	62.0	28.9	44.8	27.6	63.5	25.9
PTPE	64.2	16.2	63.1	16.0	66.2	21.6	65.3	11.3
CPE	64.8	42.3	71.6	62.1	71.8	42.2	59.2	37.4
UPE	21.8	10.5	14.2	7.7	21.0	9.6	25.0	10.6
Malignant	21.4	23.7	14.9	8.5	16.9	11.3	28.8	33.4
Miscellaneous	19.5	18.9	20.6	41.6	17.6	8.2	20.8	6.3
Transudative	9.7	5.3	6.2	2.7	8.6	4.0	13.0	5.6
Undiagnosed	19.0	8.5	12.6	3.8	18.5	8.2	25.2	7.6
Total	28.4	26.9	31.2	32.4	24.7	22.9	29.4	25.4

CTPE: confirmed pleural tuberculosis; PTPE: probable pleural tuberculosis; CPE: complicated parapneumonic effusion; UPE: uncomplicated parapneumonic effusion.

**Table 2:** Number and percentage of positive results in three prevalence tuberculosis periods (1998–2000, prevalence: 31.3%; 2001–2004, prevalence: 11.8%; and 2005–2008, prevalence: 7.4%) for two diagnostic criteria by type of pleural effusion.

	Whole period (1998–2008)				1998–2000				2001–2004				2005–2008			
	ADA ≥40 U/L		ADA ≥40 U/L + Lymphocytes ≥50%		ADA ≥40 U/L		ADA ≥40 U/L + Lymphocytes ≥50%		ADA ≥40 U/L		ADA ≥40 U/L + Lymphocytes ≥50%		ADA ≥40 U/L		ADA ≥40 U/L + Lymphocytes ≥50%	
Type of pleural effusion	No. Positive/No. investigated	(%)	No. Positive/No. investigated	(%)	No. Positive/No. investigated	(%)	No. Positive/No. investigated	(%)	No. Positive/No. investigated	(%)	No. Positive/No. investigated	(%)	No. Positive/No. investigated	(%)	No. Positive/No. investigated	(%)
CTPE	19/25	76.00	18/25	72.00	11/13	84.62	10/13	76.92	5/8	62.50	5/8	62.50	3/4	75.00	3/4	75.00
PTPE	46/48	95.83	45/48	93.75	26/28	92.86	26/28	92.86	10/10	100.00	10/10	100.00	10/10	100.00	9/10	90.00
CPE	20/29	68.97	3/29	10.34	3/5	60.00	0/5	0.00	5/8	62.50	0/8	0.00	12/16	75.00	3/16	18.75
UPE	3/92	3.26	1/92	1.09	0/16	0.00	0/16	0.00	1/31	3.23	0/31	0.00	2/45	4.44	1/45	2.22
Malignant	4/105	3.81	2/105	1.90	0/24	0.00	0/24	0.00	1/37	2.70	1/37	2.70	3/44	6.82	1/44	2.27
Miscellaneous	1/78	1.28	0/78	0.00	1/15	6.67	0/15	0.00	0/31	0.00	0/31	0.00	0/32	0.00	0/32	0.00
Transudative	0/61	0.00	0/61	0.00	0/19	0.00	0/19	0.00	0/16	0.00	0/16	0.00	0/26	0.00	0/26	0.00
Undiagnosed	1/34	2.94	1/34	2.94	0/11	0.00	0/11	0.00	0/11	0.00	0/11	0.00	1/12	8.33	1/12	8.33
Total	94/472	19.92	70/472	14.83	41/131	31.30	36/131	27.48	22/152	14.47	16/152	10.53	31/189	16.40	18/189	9.52

CTPE: confirmed pleural tuberculosis; PTPE: probable pleural tuberculosis; CPE: complicated parapneumonic effusion; UPE: uncomplicated parapneumonic effusion.

Over the whole period 1998–2008 (mean prevalence of 15.5%), the sensitivity, specificity, positive predictive value (PPV) and negative predictive value (NPV) for ADA_40_ were 89.0%, 92.7%, 69.2% and 97.9% (Table 3). The addition of LP_50_ to ADA_40_ increased the specificity of the latter for the diagnosis of TPE to 98.3%, and its PPV to 90% (Table 4).

**Table 3:** Bayesian probabilities of test parameters used. ADA ≥40 U/L.

	ADA ≥40 U/L			
	Whole period	1998–2000	2001–2004	2005–2008
Prevalence	15.5	31.3	11.8	7.4
Sensitivity	89.0	90.2	83.3	92.9
Specificity	92.7	95.6	94.8	89.7
Positive Predictive Value	69.2	90.2	68.2	41.9
Negative Predictive Value	97.9	95.6	97.7	99.4
Positive Likelihood Ratio	12.3	20.3	16.0	9.0
Negative Likelihood Ratio	0.1	0.1	0.2	0.1

**Table 4:** Bayesian probabilities of test parameters used. ADA ≥40 U/L and Lymphocytes ≥50%.

	ADA ≥40 U/L and Lymphocytes ≥50%			
	Whole period	1998–2000	2001–2004	2005–2008
Prevalence	15.5	31.3	11.8	7.4
Sensitivity	86.3	87.8	83.3	85.7
Specificity	98.3	100	99.3	96.6
Positive Predictive Value	90.0	100	93.8	66.7
Negative Predictive Value	97.5	94.7	97.8	98.8
Positive Likelihood Ratio	49.2	-	111.7	25.0
Negative Likelihood Ratio	0.1	0.1	0.2	0.2

For ADA_40_+LP_50_, sensitivity, specificity, PPV and NPV respectively were 87.8%, 100%, 100% and 94.7% for the first period (1998–2000, prevalence: 31.3%); 83.3%, 99.3%, 93.8% and 97.8% for the second period (2001–2004, prevalence: 11.8%); and 85.7%, 96.6%, 66.7% and 98.8% for the third period (2004–2008, prevalence: 7.4%).

**Figure 1:**
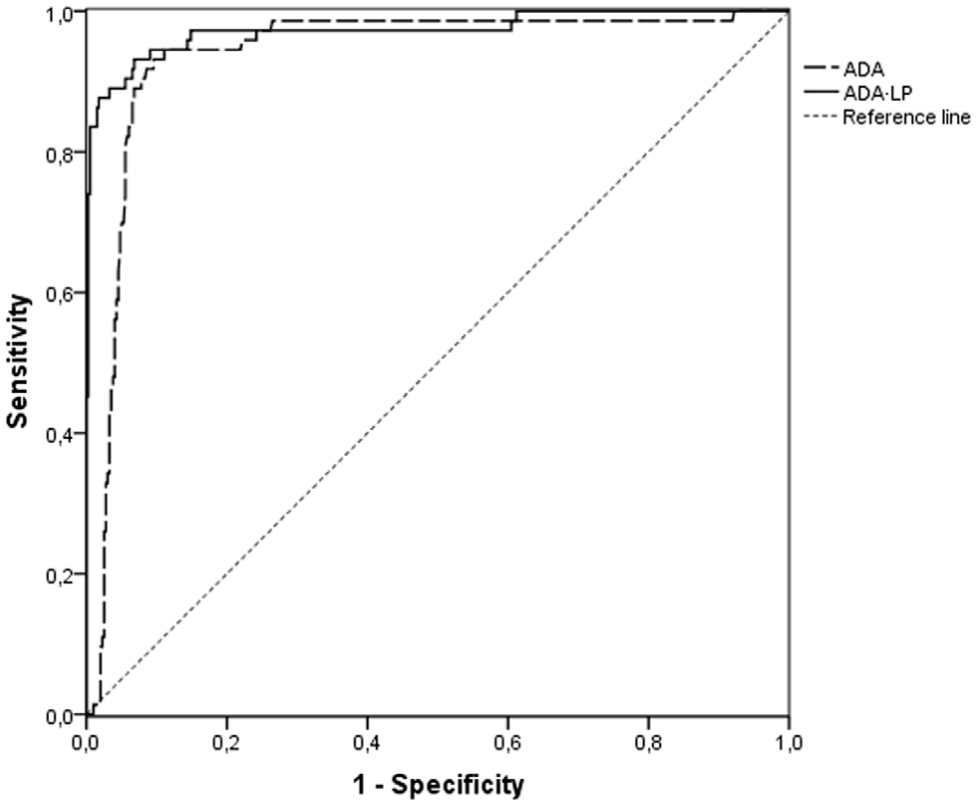
ROC curve.

For the whole period, the area under the ROC curve for ADA levels was 0.939 (95% CI 0.908–0.969) versus 0.973 (95% CI: 0.949–0.997) for ADA·LP (Figure 1), with no statistically significant differences between the three periods.

**Figure 2:**
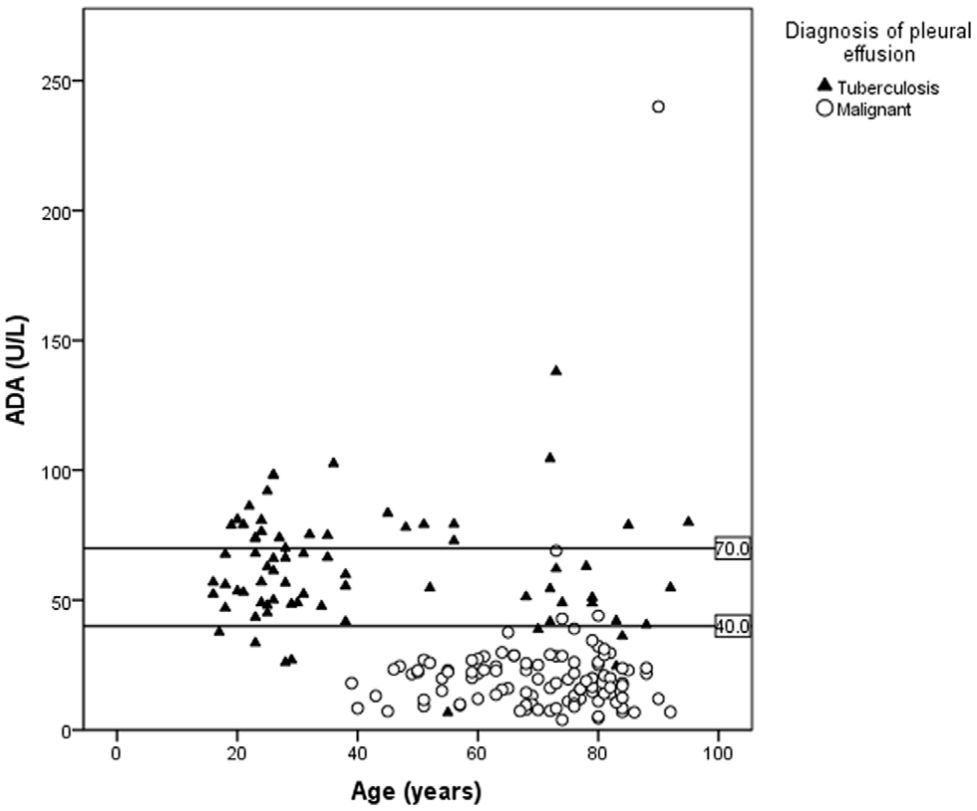
Cases of tuberculosis pleural effusion (TPE) and malignant effusion as a function of age.

Notably, all malignant effusions developed in patients over 39 years old, and just four cases out of the 105 showed an ADA level over 40 U/l, only two of them being found to be lymphocytic (1.9%). TPE, in contrast, predominated in younger people and even linear regression analysis showed that ADA activity decreased slightly with the age of the patients affected by TPE (Pearson’s correlation coefficient of −0.074). The ADA levels of 43 out of 73 patients with TPE were within the range of 40–70 U/l (Figure 2).

We observed that six out of 75 (8%) cases of lymphocytic pleural effusion had ADA>40 U/l and were of nontuberculous origin: three empyemas and one UPE (all of them after admission and drainage in another centres), along with two malignant effusions. Further, amongst the cases of CPE/empyema, 20 out of 29 (69%) were found to have an ADA level over 40 U/l, and all of them (except for the three aforementioned cases) were neutrophilic.

## Discussion

Due to the Bayesian interpretation of the well established performance of ADA for the diagnosis of TPE, it has acquired popularity as an excellent tool above all in areas of high incidence of tuberculosis [10]. Nevertheless, the role of ADA in low-intermediate prevalence settings remained uncertain, so our study comes to demonstrate that combining ADA and LP in pleural fluid the specificity and PPV of the former increase substantially, especially in low-to-intermediate prevalence settings, making this test a suitable diagnostic tool in such scenarios.

The definitive diagnosis of TPE depends on the demonstration of *Mycobacterium tuberculosis* in pleural fluid, sputum or pleural biopsy specimen, and can also be established with reasonable certainty by demonstration of granuloma in the parietal pleura. Microscopy of the pleural fluid for acid fast bacilli is positive in fewer than 5% of cases and mycobacterial culture of pleural fluid also has a low sensitivity of 35%. Closed pleural biopsy demonstrates granulomas in approximately 80% of cases, and its culture yields *Mycobacterium tuberculosis* in about 55%. Thoracoscopy offers a near 100% positive diagnostic yield on histology and 76% positive on culture [1], [11].

However, historically, since pleural biopsy is more invasive and hazardous than thoracocentesis, alternative diagnostic approaches have been extensively evaluated [2]. Unfortunately, only ADA (sensitivity 92% and specificity 89%) [12] and IFN-γ (sensitivity 89% and specificity 97%) [13] have become reliable for diagnosing TPE. The long history of the successful use of the ADA test, its simplicity, low cost, and quickly available results, make it the preferred option [1], [12], [14]. Concern regarding the absence of culture and sensitivity data when the diagnosis relies primarily on the pleural fluid ADA value has been stated, and pleural biopsy is thereby advised mostly in high mycobacterial resistance scenarios [1]. On the other hand, the addition of a culture of the (closed) pleural biopsy to that of the pleural fluid increases the overall microbiological yield from 35% to 55%, and in some authors’ opinion this invasive practise may be not justified in order to obtain such a diminished advantage [11].

Along with these biomarkers, nucleic acid amplification (NAA) tests enable direct detection of *Mycobacterium tuberculosis* in clinical specimens such as pleural fluid within hours of their collection, and can also provide information regarding antimicrobial resistance [1], [11]. However, although commercial tests were found to have a reasonably high specificity of 98% in a systematic review and meta-analysis of 40 studies, they had a low and variable sensitivity of 62% (95% CI: 43–77%) for diagnosing TPE [15]. Due to the individual limitations of each test, even sophisticated and expensive combinations of ADA, IFN-γ and NAA tests have been proposed [16].

Adenosine deaminase (ADA) is an essential enzyme in the metabolism of purine nucleosides. There are two main isoenzymes of ADA: ADA1 and ADA2. ADA 1 is ubiquitous, whereas ADA2 has been found only in monocytes and macrophages. Most of the ADA in pleural tuberculosis is ADA2 [17]; although better than total ADA for diagnosing pleural tuberculosis [18], the separation of ADA into its isoenzymes is more expensive than the basic ADA test, not readily available and its use is so far limited [1], [5], [11], [19]. The most widely accepted cut-off level of ADA for the diagnosis of TPE is 40 U/l [1], [12], [20], and an LP≥50% is conventionally accepted as a practical cut-off for lymphocytic effusion [21]. Moreover, ADA ≥70 U/l (when lymphocyte-to-neutrophil ratio is ≥0.75) has also been proposed as virtual diagnosis of TPE [11]. High ADA levels in lymphocytic pleural effusions have also been reported in rheumatoid arthritis, lymphoma, bronchoalveolar carcinoma, mesothelioma, systemic lupus erythematosus, mycoplasma and chlamydia pneumonia, psittacosis, paragonimiasis, infectious mononucleosis, brucellosis, familial Mediterranean fever, histoplasmosis and coccidioidomycosis [22]. However, apart from tuberculosis, the main disease that causes an elevated ADA is parapneumonic pleural effusion: one-third of cases of UPE and two-thirds of those of CPE/empyema may have a high ADA level, but both conditions are easily distinguished from TPE because they develop neutrophilic effusions [1], [5]. Moreover, several studies with patients suffering from nontuberculous lymphocytic pleural effusions have reported ADA levels over the diagnostic cut-off of 40 U/l in less than 3% of cases [23], [24].

Three major meta-analyses, based on 75 studies including a total of 14,505 patients, have been performed over the last decade, and these have demonstrated a uniformly high diagnostic performance of pleural ADA for TPE [12], [20], [25]. One of the major concerns regarding the sensitivity of ADA was its reliability in immunocompromised patients; however, more recent studies have demonstrated that ADA is a reliable marker of tuberculous pleurisy in HIV-positive patients, even in those with a low CD4 T-cell count [26], [27]. On the other hand, ADA levels in pleural fluid are also elevated in renal transplant recipients with tuberculous pleurisy [5].

In 1998, Valdés *et al.* were the first to propose obviating pleural biopsy for the diagnosis of tuberculous pleuritis in patients younger than 35 years of age, in areas with an intermediate (say 25%) or high prevalence of tuberculosis [28]. The limitation to young patients was related to concern regarding the more common development of malignant effusions in older patients [29] and this approach was theoretically more suitable in intermediate-to-high prevalence areas according to the Bayesian interpretation of the predictive value of ADA.

To date, almost all the studies performed concerning the diagnostic value of ADA for TPE were reported from intermediate-to-high prevalence areas, so acceptance of this test was not universal and remained contentious in countries with lower prevalence. Given this, it was considered desirable that further studies be carried out in areas of low TPE prevalence in order to confirm the suitability of this biomarker in these areas [30].

**Figure 3:**
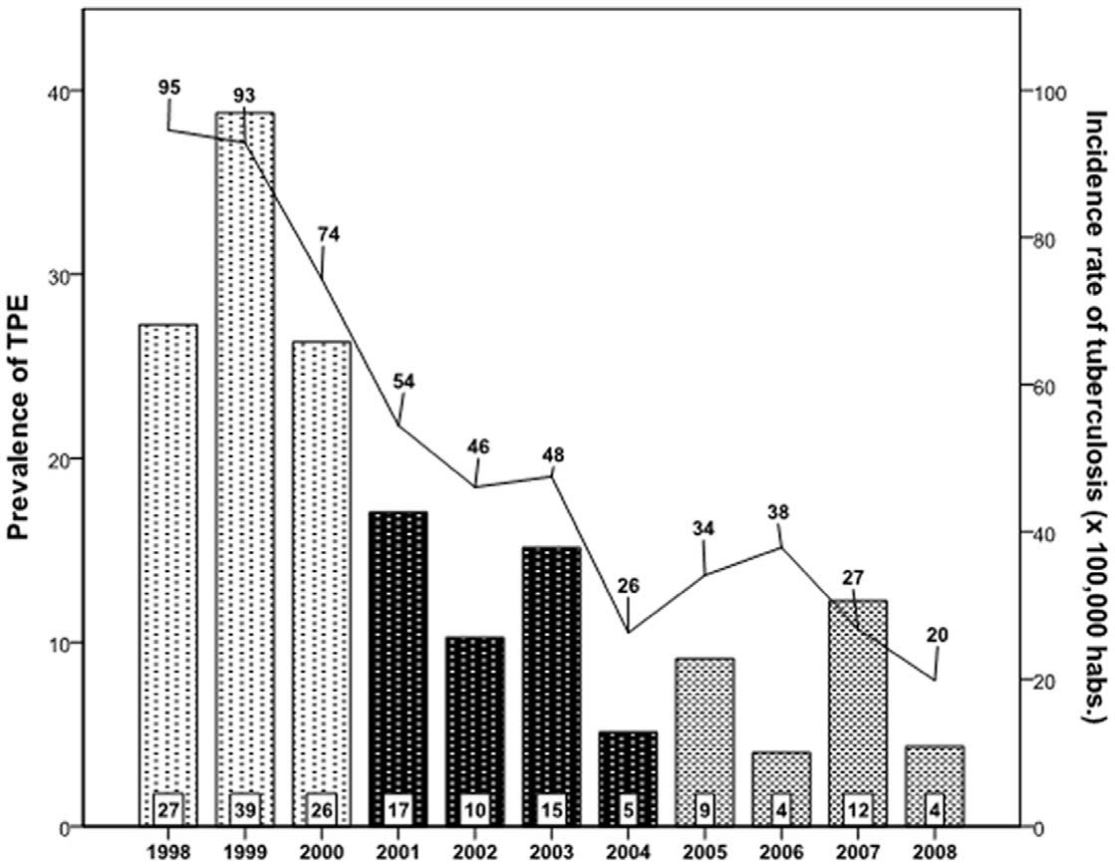
Prevalence of TPE (bars) and incidence rate of tuberculosis (line) in the Bajo Deba Area, Gipuzkoa, Basque Country (1998–2008).

Within the Multicenter Project on Tuberculosis Research performed in Spain from June 1996 to May 1997, the Bajo Deba Area was found to have the highest incidence of tuberculosis in the Basque Country (85 cases per 100,000 individuals) [31]. Subsequent implementation of a Tuberculosis Control Program by the Basque Health Service led to sustained epidemiological improvement. In the first period (1998–2000), with a 31% (intermediate-to-high) TPE prevalence scenario, we obtained a sensitivity, specificity, PPV and NPV of ADA_40_ for the diagnosis of TPE of 90.2%, 95.6%, 90.2% and 95.6%, close to values reported from previous studies performed in similar clinical settings [9], [10], [32]. During the second and third periods the prevalence of TPE declined to 11.8% and 7.8% respectively, closer to the epidemiological scenario in most Western countries [1]–[3], [12] (Figure 3). As was expected, the PPV of ADA_40_ decreased to 68.2% and 41.9% respectively, but the combination of ADA_40_ with LP_50_ increased the specificity from 94.8% to 99.3% (second period) and from 89.7% to 96.6% (third period), and simultaneously the PPV rose from 68.2% to 93.8% (second period) and from 41.9% to 66.7% (third period).

**Figure 4:**
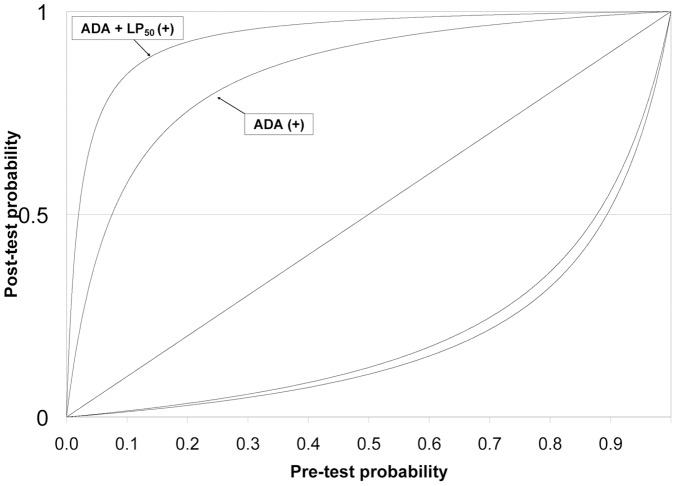
Pre-test and post-test probabilities. (+) Positive test, (-) Negative test.

In the intermediate-to-high prevalence period, both ADA_40_ and ADA_40_+LP_50_ gave very useful results. On the other hand, the addition of LP_50_ had the benefit of improving the specificity and PPV with respect to using ADA_40_ alone, especially when the prevalence was lower: the lower the prevalence, the higher this absolute increment (Tables 3 and 4). Accordingly, the performance of ADA+LP was considerably better than ADA as a single diagnostic variable, as can be seen in the post- vs. pre-test probability plots. Specifically, for rates of pleural TPE prevalence of 5%, 25% and 85% (estimated rates for low, intermediate and high prevalence countries [12]), post-test probabilities for an ADA_40_ were 41%, 81% and 99% respectively; whereas for ADA_40_+ LP_50_ they were 78%, 95% and 99% respectively (Figura 4).

Regarding the comparative study of TPE and malignant pleural effusions (Figure 2), there are some interesting findings to comment on: 1) all patients with malignant effusion in our series were over 39 years of age, and almost all of them had an ADA level <40 U/l; and 2) TPE predominated in younger patients, linear regression analysis demonstrating that ADA activity decreased with age among the patients with TPE, as previously reported by other authors [33]. Further, 43 out of 73 (59%) patients diagnosed with TPE showed an ADA level in pleural fluid of between 40 and 70 U/l. Finally, only two out of 105 patients (1.9%) with malignant pleural effusion had an ADA level and LP comparable to those theoretically attributable to TPE, so, as reported by other authors [23], [24], although always a concern, a high ADA level in nontuberculous lymphocytic pleural effusions (including malignant ones) is relatively uncommon.

Our study does have limitations: in particular, being retrospective, diagnoses based on clinical evidence were included, and though this is not optimal it is considered acceptable [16]. We note that the administration of empirical anti-tuberculosis treatment to patients with lymphocytic exudates and a high ADA level in pleural fluid when there is clinical suspicion of tuberculosis is a common practice in intermediate-to-high burden settings like our country [10]. Moreover, the universal determination of ADA level in absolutely all pleural fluid samples taken, the absence of significant differences between its mean level in the two diagnostic groups (confirmed and probable pleural tuberculosis), and the follow-up of all the patients diagnosed with TPE until complete recovery under anti-tuberculosis treatment are positive features, enhancing the validity of our study.

In conclusion, local prevalence of TPE has a more limited impact than expected (by Bayesian interpretation) on the diagnostic value of ADA when combined with LP, remaining a useful diagnostic tool in low-to-intermediate prevalence scenarios. In our experience, ADA ≥40 U/l is a highly suitable cut-off level, and in patients younger than 40 years it might constitute a virtual diagnosis of TPE when effusions are lymphocytic.
